# Mindfulness Training Improves Quality of Life and Reduces Depression and Anxiety Symptoms Among Police Officers: Results From the POLICE Study—A Multicenter Randomized Controlled Trial

**DOI:** 10.3389/fpsyt.2021.624876

**Published:** 2021-02-26

**Authors:** Marcelo Trombka, Marcelo Demarzo, Daniel Campos, Sonia B. Antonio, Karen Cicuto, Ana L. Walcher, Javier García-Campayo, Zev Schuman-Olivier, Neusa S. Rocha

**Affiliations:** ^1^Postgraduate Program in Psychiatry and Behavioral Sciences, Federal University of Rio Grande do Sul (UFRGS), Porto Alegre, Brazil; ^2^Department of Psychiatry, Hospital de Clínicas de Porto Alegre, Porto Alegre, Brazil; ^3^Innovations and Interventions for Quality of Life Research Group, Porto Alegre, Brazil; ^4^Clinical Research Center, Hospital de Clínicas de Porto Alegre, Porto Alegre, Brazil; ^5^Department of Psychiatry, Harvard Medical School, Boston, MA, United States; ^6^Cambridge Health Alliance, Center for Mindfulness and Compassion, Cambridge, MA, United States; ^7^Mente Aberta, Brazilian Center for Mindfulness and Health Promotion, Department of Preventive Medicine, Federal University of São Paulo (UNIFESP), São Paulo, Brazil; ^8^Department of Psychology and Sociology, University of Zaragoza, Huesca, Spain; ^9^Grupo de Investigación en Salud Mental en Atención Primaria, Miguel Servet University Hospital, Zaragoza, Spain

**Keywords:** mindfulness, police officer, quality of life, depression, anxiety, religiosity, well-being, self-compassion

## Abstract

**Background:** Police officers' high-stress levels and its deleterious consequences are raising awareness to an epidemic of mental health problems and quality of life (QoL) impairment. There is a growing evidence that mindfulness-based interventions are efficacious to promote mental health and well-being among high-stress occupations.

**Methods:** The POLICE study is a multicenter randomized controlled trial (RCT) with three assessment points (baseline, post-intervention, and 6-month follow-up) where police officers were randomized to mindfulness-based health promotion (MBHP) (*n* = 88) or a waiting list (*n* = 82). This article focuses on QoL, depression and anxiety symptoms, and religiosity outcomes. Mechanisms of change and MBHP feasibility were evaluated.

**Results:** Significant group × time interaction was found for QoL, depression and anxiety symptoms, and non-organizational religiosity. Between-group analysis showed that MBHP group exhibited greater improvements in QoL, and depression and anxiety symptoms at both post-intervention (QoL *d* = 0.69 to 1.01; depression *d* = 0.97; anxiety *d* = 0.73) and 6-month follow-up (QoL *d* = 0.41 to 0.74; depression *d* = 0.60; anxiety *d* = 0.51), in addition to increasing non-organizational religiosity at post-intervention (*d* = 0.31). Changes on self-compassion mediated the relationship between group and pre-to-post changes for all QoL domains and facets. Group effect on QoL overall health facet at post-intervention was moderated by mindfulness trait and spirituality changes.

**Conclusion:** MBHP is feasible and efficacious to improve QoL, and depression and anxiety symptoms among Brazilian officers. Results were maintained after 6 months. MBHP increased non-organizational religiosity, although the effect was not sustained 6 months later. To our knowledge, this is the first mindfulness-based intervention RCT to empirically demonstrate these effects among police officers. Self-compassion, mindfulness trait, and spirituality mechanisms of change are examined.

**Clinical Trial Registration:** www.ClinicalTrials.gov. identifier: NCT03114605.

## Introduction

Policing is a high-stress occupation (1, 2), ranking as one of the most psychologically dangerous professions worldwide (3, 4). Different from other vulnerable occupations, officers repeatedly face dangerous, unpredictable, and potentially traumatic situations (e.g., exposure to human misery and death, shooting, critical incidents, and life-changing errors with emotional cost). Dealing with verbal and physical aggression, having to use force to restrain, giving evidence in court, and high-speed driving are examples of operational stressors (4). Organizational stressors often include hierarchical and bureaucratic structures which can perpetuate discrimination, sexism, and racism; poor communication; long and irregular shifts; new recruit hazing and cultural assimilation; hostile public image; and inadequate resources (4–6). Exposure to acute and chronic stressors are associated with a myriad of negative consequence for officers' mental health, physical health, and quality of life (QoL).

When compared to the general population, officers exhibit higher rates of depression (7), anxiety (8), burnout (9), alcohol abuse (10), post-traumatic stress disorder (PTSD) (11), and suicide (12). Police perceived work stress predicts depression and anxiety symptoms (7, 13). For instance, the prevalence of depression among officers is nearly twice as high as the general population (14). Half of all completed suicides are related to depressive and other mood disorders (15). Anxiety disorders more than double the risk of suicide attempts, and a combination of depression and anxiety greatly increases the risk by 17 (16, 17). Recent data show that in the USA, more officers die from suicide than in the line of duty (18). Poor mental health can be deleterious to physical health. Indeed, officers have elevated risks for diabetes, obesity, cardiovascular disease, and sudden cardiac death (19–21). Brazilian Police mental health scenario is also alarming, with a high prevalence of stress, depression, anxiety, and PTSD (6, 22–24). Crime rate in Brazil is high and exposure to violence is associated with occupational stress, burnout, and suicide (23, 25–28). The 2019 Brazilian Public Annual Report evidenced the same pattern as the USA: more officers died from suicide than at work (29).

Work-related stress, accompanied by a culture where display of emotion is often viewed as a sign of weakness (30), pays a toll on multiple work and life dimensions. Officers experiencing high levels of stress and fatigue are less efficient and prone to absenteeism, uncontrolled anger toward suspects, impaired judgment and decision-making (31, 32); increased implicit racial bias (33), including the decision to shoot (34); and to use excessive force (35, 36). Disruptive family relationships and marital troubles are another facet of the pernicious consequences of stress (37, 38). QoL is a broad-ranging concept, affected in a complex way by the person's physical health, psychological state, level of independence, social relationships, and their relationships to salient features of their environment (39). Perceived stress levels are negatively associated with QoL and explain up to 34% of its variability (40). Two studies evidenced significant associations between Brazilian police stress and QoL deficits in the social, affective, professional, and health areas (6, 23). Extensive literature associate religiosity and its dimensions (i.e., organizational, non-organizational, intrinsic) with psychological health and QoL (41–43). It is negatively related to stress among officers and can be an effective coping tool for occupational stress (44, 45).

The last two decades have seen a dramatic increase in the scientific research and popular interest about mindfulness. Mindfulness can be defined as an innate meta-awareness capacity to attend to present-moment experience while avoiding entanglement in cognitive biases with an orientation of curiosity, openness, and acceptance (46). It can be enhanced by simple meditative attentional practices, as well as through structured training programs such as mindfulness-based interventions (MBIs). MBIs are group-based weekly classes composed of meditation practices, such as the body scan, mindful movement, and sitting and walking meditation; experiential inquiry-based learning process; and exercises to develop insight (47). MBIs implicitly teach self-compassion, which is composed of three interacting components: self-kindness (i.e., being kind and understanding toward oneself in instances of perceived inadequacy or suffering vs. self-judgment), a sense of common humanity (i.e., perceiving one's experiences as part of the larger human experience vs. isolation), and mindfulness itself (i.e., holding one's painful thoughts and feelings in balanced awareness vs. over identification with suffering or avoidance/disconnection) (48). Mindfulness training (MT) may regulate how the individual appraises stress and increase secondary appraisals of approach-oriented coping resources, thus reducing stress reactivity. A process of enhanced self-regulation is established by synergistic systems of attention/cognitive control, emotional regulation, and self-related processes (49, 50).

Meta-analyses indicate that MBIs decrease stress (51), depression and suicidal ideation (52, 53), anxiety and burnout (53, 54), and increases QoL (55) and spirituality (56). MBIs have been shown to be feasible and to lead to improved health outcomes in non-clinical populations at the workplace (57), including several high-stress occupations, such as the military (58–62), healthcare providers (63), firefighters (64), and inner-city teachers (65). Published in 2016, a single-arm pilot study pioneered the field of MT within law enforcement officers (LEOs), suggesting positive effects following mindfulness-based resilience training (MBRT)—an 8-week mindfulness-based stress reduction (MBSR) adaptation designed to enhance resilience for LEOs in the context of stressors inherent to policing—on outcomes such as perceived stress, burnout, emotional intelligence, and mental and physical health (66). Since then, three other trials have replicated and extended those benefits (67–69).

With the development of positive psychiatry, specialty scope incorporates—along with the treatment of mental illness—psychosocial interventions targeting those at high risk of developing mental or physical illness, and goals encompass increased well-being and positive psychosocial characteristics (e.g., resilience, spirituality, religiosity) (70). Two reviews concluded that randomized controlled trials (RCTs) are needed to clarify the role of psychosocial interventions for stress management and mental health/well-being promotion among police officers (71, 72). In light of these findings, the burden of high-stress levels and mental health problems epidemic in the police, plus aforementioned MBIs' salutary effects, further empirical evaluation of MBIs' impact on that vulnerable population is needed. The POLICE (im*P*act *O*f a mindfu*L*ness-based *I*ntervention on burnout and quality of life in poli*CE* officers) study is a multicenter RCT evaluating the efficacy of mindfulness-based health promotion (MBHP) to promote police officers' QoL and mental health at post-intervention and after 6 months. As designated at the research protocol manuscript (73), our hypothesis is that officers allocated to MBHP, compared with a waiting list (WL), will show enhancement on QoL and reduction on burnout symptoms (primary outcomes), besides improvement on several mental health measures (secondary outcomes). We also investigated primary outcomes of potential mechanisms of change through mediation and moderation analysis. Mediators clarify how/why intervention works, while moderators identify whom or under what conditions interventions have effects (74). The POLICE study design overcomes previous studies' limitations such as absence of control group/follow-up or small sample sizes.

## Materials and Methods

A detailed description of POLICE study Materials and Methods is available at the research protocol manuscript (73).

### Design

**Figure 1:**
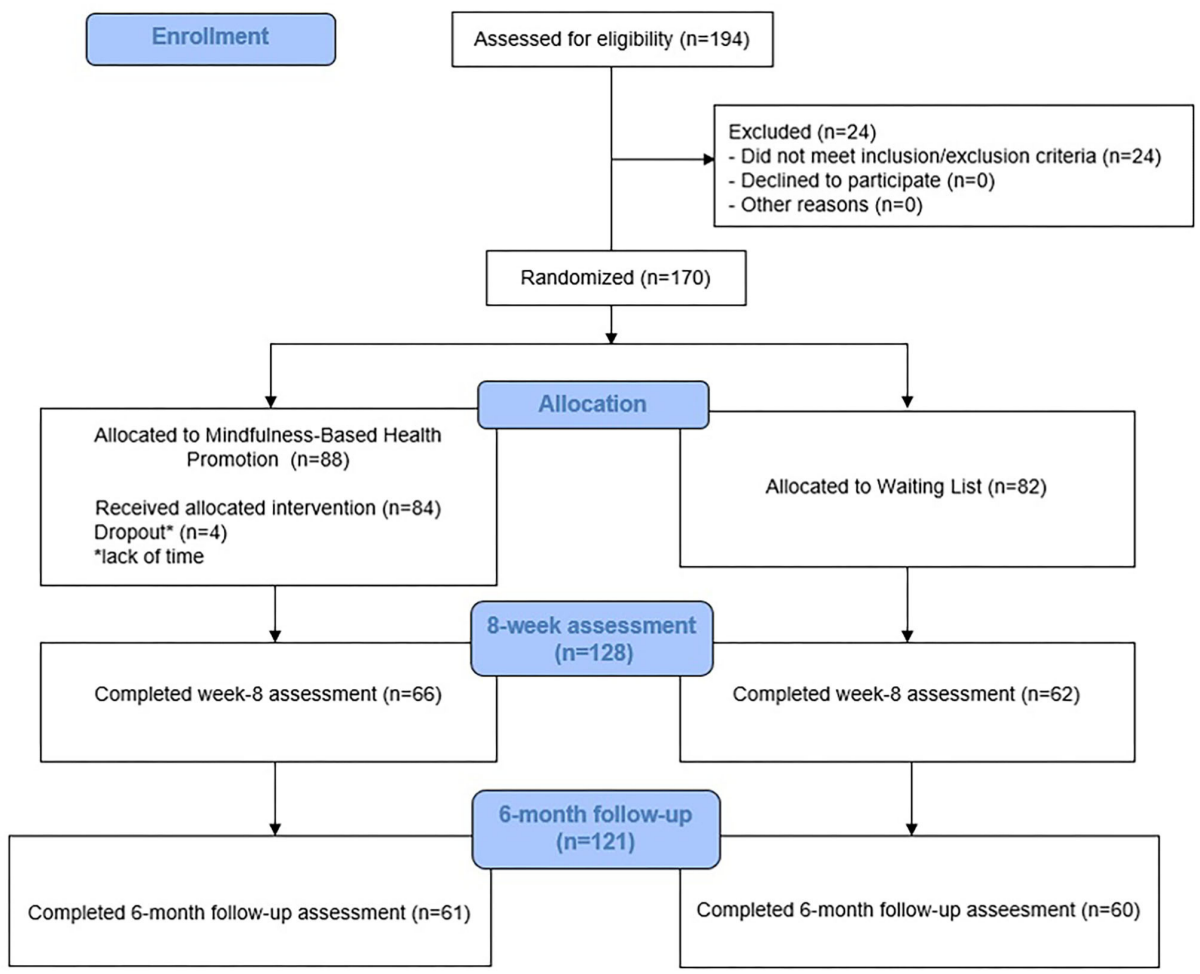
Consort diagram.

The POLICE study was a multicentric, parallel, two-armed RCT with three assessment points: baseline, post-intervention, and 6-month follow-up (73). Participants were randomized to MBHP—an 8-week MBI—or a WL control group. The clinical trial protocol was prepared in accordance with SPIRIT 2013 statement (75) and was approved by the centers' ethical committees. Figure 1 shows the CONSORT flow diagram through the study.

### Participants and Settings

Police officers working at two major Brazilian cities were recruited from 2016 to 2018.

(1) Civil Police. The study center in Porto Alegre was located at the Police Hall of the State of Rio Grande do Sul. Civil Police main duties are to oversee public order and security, adopt measures necessary to avoid danger or injury to persons and public or private property, and ensure the administration of criminal offenses, including execution of arrest warrants and requisitions requested by the judicial branch.

(2) Civil Guard. The study center in São Paulo was located at the headquarters of Unifesp Santo Amaro. Civil Guard fundamental duties include the protection of goods, services, and municipal facilities, as well as patrolling areas and preventive policing.

Participants meeting the following inclusion criteria were enrolled: (1) active police officers; (2) 21–65 years old; (3) availability to attend eight sessions; (4) willingness to participate voluntarily. Exclusion criteria included (1) previous involvement in any MBI or regular mindfulness practice over the last 3 months, or one of the following diagnosis, assessed by the *Mini International Neuropsychiatric Interview (MINI)*: (2) major depressive episode (current); (3) manic or hypomanic episode (current); (4) psychotic syndrome (current or past); (5) substance use disorder (past 12 months, except tobacco); (6) risk of suicide.

### Procedures

The POLICE study was advertised within the two Police Institutions using the internet, social media, and posters. Officers who showed interest contacted the research team by phone or email and were initially screened. Potential participants had a face-to-face interview scheduled where they were provided detailed information about the study and inclusion/exclusion criteria were assessed. Eligible volunteers filled the informed consent form and were randomly assigned to MBHP or WL by an independent researcher who were unaware of the characteristics of the study and was not involved in the trial or had access to study data. Randomization was implemented using sealed envelopes. Officers allocated to the WL group did not receive any intervention for 6 months. After the 6-month assessment, MBHP was offered for all participants randomized to the WL group. Officers received authorization from the Police Institutions to attend the program which happened during working hours. Due to the nature of the intervention, the POLICE study was single-blind—the outcomes assessment was blind, albeit participants were aware of their group assignment. While we initially planned to randomize 160 participants (allowing for up to 20% loss to follow-up) to detect a moderate effect size (Cohen's *d* = 0.05) with a power of 0.80 and an alpha of 0.05, from the 194 participants screened, 170 officers met enrollment criteria and were randomized. We defined participants as external police officers if during the study they were exerting operational activities and as internal police officers if their contact with civilians was done inside police agencies or they were assigned to administrative roles.

### Intervention

MBHP (76) is an 8-week MBI based on the MBSR model (77). It was adapted to suit the context and needs of primary healthcare and national and local health systems, developing a framework that supports the learning process to individuals from distinctive cultural and educational backgrounds. MBHP was designed to address human universal vulnerabilities, not focusing on any specific health condition. It has been applied since 2009 by Mente Aberta—Brazilian Center for Mindfulness and Health Promotion, and by the University of Zaragoza in Spain. There is a strong emphasis on informal mindfulness practices, such as walking, eating, exercising, talking, and doing housework. Concepts of radical acceptance (78), values clarification (79), and positive psychology (80) are heavily emphasized. Session six is conducted in silence for the purpose of deepening the practice. Besides MBSR core practices, psychoeducational activities and practices from mindfulness-based cognitive therapy (MBCT) and Breathworks curriculum are introduced (i.e., 3-min breathing space; “primary and secondary suffering;” “hello-thanks-goodbye thought”) (81, 82). Compassion training techniques are also included (equanimity in the condition of human suffering, receiving affection and showing affection to oneself). This rigorous training follows the British and Brazilian guidelines for good practices of mindfulness (83, 84). MBHP groups started with an average of 21 officers. One interventionist led the MBHP groups in Porto Alegre and one led the MBHP groups in São Paulo. MBHP core curriculum content is described in Table 1.

**Table 1:** Mindfulness-based health promotion (MBHP) core curriculum content.

**Session**	**Didactive teaching**	**Practices**	**Homework**
1. Mindfulness: leaving the automatic pilot	Presentation and aims Stress and dispersion What is and what is not mindfulness Characteristics, attitudes, and motivation Introduction to practice diaries	Eating a raisin Simple mindfulness exercises (listening to sounds, areas of contact with your body, sensations of your feet touching the floor) Body scan 9-dots exercise	Body scan Attention for routine activity Eating one meal mindfully Mindfulness diary
2. Mindfulness in the body	What to do with the body What to do with the mind Breathing Information on posture Preconceptions/fears/challenges in meditation	Mindfulness in breathing Body scan Primary and secondary suffering	Body scan Mindfulness in breathing Awareness of pleasant events (diary) Attention for routine activity Habit changing
3. Mindfulness in movement	Importance of the body How mindfulness works Cognitive defusion	Mindful walking Mindfulness in breathing Hello-thanks-goodbye thought	Body scan Mindfulness in breathing Mindful walking Awareness of unpleasant events (diary) Attention for routine walking
4. Expanding mindfulness	Doing mode/being mode Reinforcing daily practice Obstacles to the practice Self-compassion	Mindful movements Mindfulness in breathing, sensations, sounds, and thoughts 3-min breathing space Mindful walking	Body scan Mindfulness in breathing Mindful walking 3-min breathing space Awareness of communication difficulties (diary)
5. Dealing with difficulties with acceptance	Importance of acceptance Values and committed action Meaning of life Hedonic and eudaimonic well-being	Mindful movements Mindfulness of thoughts 3-min breathing space in doubles	Practice of choice 3-min breathing space during stress
6. Mindfulness in silence	All practices in silence	Mindful movements Body scan Sitting meditation Mindful walking	Practice of choice Mindfulness conversation
7. Mindfulness and compassion	What is and what is not compassion Biological basis Ways of training compassion Fear of compassion in western society	Loving kindness (for oneself and others) Sitting meditation Mindful movements	Practice of choice Loving kindness (for oneself and others) Attention for self-compassion in routine activity
8. Mindfulness for life	Mindfulness in daily life Recommendations for long-term practice (personal value-based practice)	Connecting with personal values and life purposes Loving kindness (for oneself and others) Mindfulness poetry	Daily life mindfulness incorporation

### Assessments and Outcomes

The POLICE study had three assessment points, which occurred during the 2 weeks before the intervention, the 2 weeks following the intervention, and 6 months after the intervention. Participants allocated to the WL filled the surveys on the same timeline. Surveys were answered on the Survey Monkey Software (http://surveymonkey.com). When computers or tablets were not available, participants answered using pen and paper and the data were transcribed via single entry to the password-protected software. Primary and secondary outcomes were previously reported (73). This article will specifically report the findings related to QoL, anxiety and depression symptoms, and religiosity. We will also address MBHP feasibility and the possible role of mediators (i.e., mindfulness trait, decentering, self-compassion, and spirituality) and moderators (i.e., sex, age, number of sessions, mindfulness trait, decentering, self-compassion, and spirituality) to post-intervention QoL improvement as formerly proposed (73). Because the Connor-Davidson-25 Scale of Resilience (CD-RISC-25) was not applied to all participants, we excluded resilience from our mediation and moderation analysis.

#### Primary Outcome

##### World Health Organization Quality of Life-BREF (WHOQOL-BREF)

Twenty-six-item instrument that produces scores for four domains related to QoL: physical health, psychological, social relationships, and environment, besides overall QoL and general health facets. WHOQOL-BREF Portuguese version exhibits strong psychometric properties (85). Cronbach's α in the present sample was physical health, α = 0.79; psychological, α = 0.84; social relationships, α = 0.79; and environment, α = 0.76.

#### Secondary Outcomes

##### Hospital Anxiety and Depression Scale (HADS)

Fourteen-item scale that quantifies the severity of anxiety (HADS-A) and depressive symptoms (HADS-D) in community and hospital settings. HADS Portuguese version shows good psychometric properties (86). Cronbach's α in the present sample was 0.81 for HADS-A and 0.78 for HADS-D.

##### Duke University Religion Index (DUREL)

Five-item instrument capturing three dimensions of religiosity: organizational religiosity (OR), non-organizational religiosity (NOR), and intrinsic religiosity (IR). The NOR subscale measures private religious activities (e.g., prayer, meditation, Scripture study), while the OR subscale involves public religious activities. IR subscale assesses the degree of personal religious commitment or motivation, pursuing religion as an ultimate end in itself (43). DUREL Portuguese version presents high internal consistency and discriminant validity (87). Cronbach's α in the present study was 0.88.

### Mechanisms of Change

#### Mindful Attention Awareness Scale (MAAS)

Fifteen-item scale designed to assess a core characteristic of dispositional mindfulness. MAAS Portuguese version has adequate reliability and validity (88). Cronbach's α in the present study was 0.96.

#### Self-Compassion Scale (SCS)

Twenty-six-item instrument measuring six components of self-compassion. Portuguese SCS version exhibits good psychometric properties (89). Cronbach's α in the present study was 0.81.

#### WHOQOL-SRPB-BREF

Thirty-four-item scale evaluating QoL domains of spirituality, religiosity, and personal beliefs. WHOQOL-SRPB-BREF Portuguese version shows strong psychometric properties (90). Cronbach's α in the present study was 0.88.

### Data Analysis

Group differences at baseline on demographic data and outcome variables were evaluated using χ^2^-tests for categorical variables and Student's *t*-test for continuous data. Intention-to-treat (ITT) mixed-models analyses without any *ad hoc* imputation were used to account for missing data with the restricted maximum likelihood estimation (REML) (91), rather than repeated measures ANOVA described in the original protocol based on the authors' recommendation and due to the amount of missing data. The mixed-model approach is appropriate for RCTs with multiple time points and pre-only to post-only designs, it does not assume that the last measurement is stable, it does not involve any substitution of missing values with supposed or estimated values, it is conducted using all available observations (92, 93), and it is robust to violations of distributional assumptions (94). A linear mixed model for each primary and secondary outcome measure was implemented with *time* (pre, post, and follow-up) as within-group factor and *group* (WL and MBHP) as between-group factor using the MIXED procedure with a random intercept for subject. An identity covariance structure was specified to model the covariance structure of the intercept. Wald statistic (or *Z*-test) was conducted to test the residual error variance estimation and the null hypothesis of homogeneity of residuals (95, 96). Significant effects were followed up with pairwise contrasts adjusted by Bonferroni correction. These statistical tests have shown their robustness regardless of violations of the required assumptions when group sizes are equal (97). Results are reported in line with conventional ANOVA, as mixed-model repeated measures, according to studies that used this approach for repeated measures designs (98, 99). Effect sizes (Cohen's *d*) and the 95% CI were calculated for within- and between-group comparisons, based on Botella and Sanchez-Meca, and Cumming and Calin-Jageman recommendations (100, 101). Mediation hypothesis was tested by bootstrap regression analysis using the Preacher and Hayes approach (PROCESS) (Model 4) (102). The pre-to-post changes in mindfulness (MAAS), decentering (EQ), self-compassion (SCS), and spirituality (WHOQOL-SRPB-BREF) were included as proposed mediators between group and QoL outcome. Separate moderation analyses were conducted for WHOQOL-BREF using the Bootstrapping PROCESS (model 1). Independent moderation models were tested for each proposed mediator: sex, age, number of sessions, and pre-to-post changes on mindfulness (MAAS), decentering (EQ), self-compassion (SCS), and spirituality (WHOQOL-SRPB-BREF). Significant moderation was followed up by significant interaction between the independent variable (X) (group: WL vs. MBHP) and the dependent variable (Y) (pre-to-post changes in WHOQOL-BREF). Statistical analyses were performed using the IBM SPSS version 23 for Windows.

## Results

### Baseline Data and Participant Characteristics

Descriptive statistics and sociodemographic characteristics of participants are shown in Table 2. No statistically significant differences between groups were found on any sociodemographic data, or on primary and secondary outcomes at baseline.

**Table 2:** Sociodemographic data.

**Variable**	**Total sample** ** (*n* = 170)**	**WL** ** (*n* = 82)**	**MBHP** ** (*n* = 88)**	**Statistics** ** (WL vs. MBHP)**
Age	42.26 (7.71) (range = 24–60)	42.46 (7.95) (range = 24–60)	42.07 (7.53) (range = 24–59)	t_(168)_ = 0.33 *p* = 0.74
Sex				χ^2^_(1)_ = 0.937 *p* = 0.33
Male	43 (25.3%)	18 (22%)	25 (28.4%)	
Female	127 (74.7%)	64 (78%)	63 (71.6%)	
Educational status				χ^2^_(1)_ = 2.238 *p* = 0.135
High school	15 (8.8%)	10 (12.2%)	5 (5.7%)	
University	155 (91.2%)	72 (87.8%)	83 (94.3%)	
Type of work				χ^2^_(1)_ = 0.377 *p* = 0.539
Internal	85 (50%)	43 (52.4%)	42 (47.7%)	
External	85 (50%)	39 (47.6%)	46 (52.3%)	
Stable partner				χ^2^_(1)_ = 0.005 *p* = 0.94
Yes	121 (71.2%)	57 (69.5%)	64 (72.7%)	
No	44 (25.9%)	21 (25.6%)	23 (26.1%)	
Missing data	5 (2.9)	4 (4.9)	1 (1.1%)	

*Means and SDs are represented for age (years). WL, waiting list control group; MBHP, mindfulness-based health promotion*.

### Feasibility

We randomized 87.6% (*n* = 170 out of 194) of officers assessed for eligibility. Due to reported lack of time, four individuals did not start the intervention. Participants who were randomized to the MBHP arm and started the intervention (*n* = 84) attended a mean of 6.44 (SD 2.14) of eight weekly sessions, 82.1% (*n* = 69) attended at least four sessions, and 69% (*n* = 58) at least six sessions. Eight-week assessment was completed by 75.3% (*n* = 128) and 6-month follow-up was answered by 71.2% (*n* = 121) of the enrolled sample.

### Change in Primary and Secondary Outcomes From Pre- to Post-intervention and 6-Month Follow-Up

#### Primary Outcome: Quality of Life (WHOQOL-BREF)

Results for the WHOQOL-BREF showed a significant group × time interaction effect in all QoL domains—physical health [*F*_(2,252.59)_ = 13.83; *p* < 0.001], psychological [*F*_(2,245.89)_ = 20.36; *p* < 0.001], social relationships [*F*_(2,248.25)_ = 9.02; *p* < 0.001], and environment [*F*_(2,250.096)_ = 15.20; *p* < 0.001], the overall QoL facet [*F*_(2,251.98)_ = 7.51; *p* < 0.01), and general health facet [*F*_(2,254.216)_ = 5.10; *p* < 0.05]. Within-group comparisons revealed a significant pre-to-post and pre-to 6-month follow-up changes in MBHP group for physical health [*F*_(2,251.67)_ = 18.65; *p* < 0.001], psychological [*F*_(2,244.84)_ = 19.12; *p* < 0.001], social relationship [*F*_(2,247.02)_ = 12.72; *p* < 0.001], environment [*F*_(2,248.77)_ = 18.67; *p* < 0.001], overall QoL [*F*_(2,249.20)_ = 9.13; *p* < 0.001], and general health [*F*_(2,252.38)_ = 15.16; *p* < 0.001]. No significant changes were found in WL group except for pre-to-post reductions on the psychological domain (Table 3).

**Table 3:** Means, SDs, and within-group effect sizes for primary and secondary outcomes at pre-, post-intervention, and 6-month follow-up.

	**Waiting list**					**Mindfulness-based health promotion**				
**Outcome**	**Pre** ** (*N* = 82)**	**Post** ** (*N* = 62)**	**FU** ** (*N* = 60)**	**Pre vs. post** ** Mean dif.; d (95% CI)**	**Pre vs. FU** ** Mean dif.; d (95% CI)**	**Pre** ** (*N* = 88)**	**Post** ** (*N* = 66)**	**FU** ** (*N* = 61)**	**Pre vs. post** ** Mean dif.; d (95% CI)**	**Pre vs. FU** ** Mean dif.; d (95% CI)**
**WHOQOL-BREF**										
Physical health	14.49 (2.51)	14.00 (3.12)	14.32 (2.83)	0.40 d = −0.19 (−0.41, 0.02)	0.24 d = −0.07 (−0.30, 0.07)	14.70 (2.44)	16.26 (2.20)	16.12 (2.19)	1.52^***^ d = 0.63 (0.37, 0.89)	1.28^***^ d = 0.57 (0.33, 0.82)
Psychological	14.60 (2.49)	13.87 (3.18)	14.42 (2.53)	0.80^**^ d = −0.13 (−0.34, 0.08)	0.25 d = −0.03 (−0.21, 0.15)	14.83 (2.40)	16.47 (1.79)	16.16 (2.24)	1.42^***^ d = 0.66 (0.42, 0.91)	1.09^***^ d = 0.54 (0.29, 0.78)
Social relationships	13.89 (3.20)	13.43 (3.77)	13.84 (2.92)	0.38 d = −0.14 (−0.37, 0.08)	0.19 d = −0.03 (−0.30, 0.23)	14.12 (3.14)	15.79 (2.26)	15.50 (2.89)	1.58^***^ d = 0.53 (0.28, 0.77)	1.25^**^ d = 0.43 (0.20, 0.67)
Environment	13.50 (2.44)	13.14 (2.75)	13.78 (2.34)	0.38 d = −0.15 (−0.36, 0.07)	0.35 d = 0.11 (−0.12, 0.35)	13.31 (2.10)	14.72 (1.69)	14.68 (1.96)	1.26^***^ d = 0.66 (0.40, 0.93)	1.23^***^ d = 0.61 (0.39, 0.90)
Overall quality of life	14.86 (3.42)	14.41 (3.66)	14.40 (4.04)	0.38 d = −0.13 (−0.34, 0.09)	0.53 d = −0.13 (−0.43, 0.16)	14.95 (3.12)	16.49 (1.80)	16.72 (1.80)	1.40^**^ d = 0.49 (0.16, 0.81)	1.60^**^ d = 0.56 (0.23, 0.89)
General health	12.74 (3.80)	12.97 (4.51)	13.67 (3.83)	0.11 d = 0.06 (−0.17, 0.29)	0.77 d = 0.24 (−0.03, 0.52)	13.50 (3.70)	15.69 (3.11)	15.74 (2.72)	2.04^***^ d = 0.58 (0.34, 0.83)	1.93^***^ d = 0.60 (0.33, 0.86)
**HADS**										
Anxiety	7.06 (3.43)	7.55 (3.62)	7.68 (3.91)	0.49 d = 0.14 (−0.09, 0.37)	0.77 d = 0.18 (−0.09, 0.44)	6.80 (3.75)	5.10 (3.01)	5.82 (3.31)	1.63^***^ d = −0.44 (−0.69, −0.21)	1.61^***^ d = −0.26 (−0.52, 0.00)
Depression	5.68 (3.48)	6.78 (3.98)	7.14 (3.74)	1.04^*^ d = 0.31 (0.09, 0.54)	1.52^**^ d = 0.41 (0.16, 0.67)	5.45 (3.22)	3.56 (2.52)	4.74 (4.14)	1.74^*^ d = −0.58 (−0.87, −0.29)	0.59 d = −0.21 (−0.52, 0.08)
**DUREL**										
Organizational religiosity	3.25 (1.51)	3.20 (1.68)	3.26 (1.47)	0.11 d = −0.03 (−0.18, 0.12)	0.01 d = 0.01 (−0.19, 0.20)	3.40 (1.43)	3.32 (1.37)	3.46 (1.47)	0.09 d = −0.06 (−0.19, 0.08)	0.07 d = 0.04 (−0.15, 0.23)
Non-organizational religiosity	3.70 (1.64)	3.50 (1.76)	3.52 (1.63)	0.28 d = −0.12 (−0.32, 0.08)	0.17 d = −0.11 (−0.33, 0.11)	3.67 (1.63)	4.03 (1.60)	3.75 (1.54)	0.31 d = 0.21 (0.04, 0.39)	0.02 d = 0.05 (−0.17, 0.23)
Intrinsic religiosity	11.16 (3.44)	11.63 (3.50)	11.48 (3.17)	0.24 d = 0.13 (−0.01, 0.28)	0.23 d = 0.09 (−0.10, 0.27)	11.49 (3.15)	11.88 (3.34)	12.13 (3.29)	0.44 d = 0.12 (−0.01, 0.23)	0.39 d = 0.20 (0.05, 0.36)
Overall DUREL	18.11 (5.85)	18.32 (6.15)	18.26 (5.65)	0.19 d = 0.04 (−0.09, 0.16)	0.05 d = 0.03 (−0.13, 0.18)	18.55 (5.40)	19.23 (5.38)	19.34 (5.35)	0.65 d = 0.12 (0.01, 0.24)	0.24 d = 0.14 (0.00, 0.29)

*Means and SDs are represented for primary and secondary outcomes. Pre, pre-treatment; Post, post-intervention; FU, 6-month follow-up; d, Cohen's d; WHOQOL-BREF, World Health Organization Quality of Life–BREF; HADS, Hospital Anxiety and Depression Scale; DUREL, Duke University Religion Index*.

* *p < 0.05*,

** *p < 0.01*,

*** *p < 0.001. Means differences are described with absolute values and were calculated using estimated marginal means adjusted by Bonferroni for multiple comparisons. P-values of the Wald test were < 0.05 for primary and secondary outcomes*.

Between-group comparisons showed significant differences between groups, indicating higher scores in the MBHP group at post and 6-month follow-up compared with the WL control group across all domains and facets (Table 4).

**Table 4:** Between-group comparisons at post and 6-month follow-up.

**Outcome**	**Pre (MBHP vs. WL)**	**Post (MBHP vs. WL)**		**FU (MBHP vs. WL)**	
	***F*-values**	***F*-values**	**d (95% CI)**	***F*-values**	**d (95% CI)**
**WHOQOL-BREF**					
Physical health	*F*_(1, 262)_ = 0.20	*F*_(1, 320)_ = 23.62^***^	0.84 (0.48, 1.20)	*F*_(1, 330)_ = 14.86^***^	0.71 (0.34, 1.07)
Psychological	*F*_(1, 241)_ = 0.28	*F*_(1, 298)_ = 33.72^***^	1.01 (0.64, 1.38)	*F*_(1, 309)_ = 13.38^***^	0.72 (0.36, 1.09)
Social relationships	*F*_(1, 262_) = 0.25	*F*_(1, 324)_ = 17.53^***^	0.76 (0.40, 1.12)	*F*_(1, 334)_ = 9.81^**^	0.57 (0.20, 0.93)
Environment	*F*_(1, 257)_ = 0.54	*F*_(1, 317)_ = 13.95^***^	0.69 (0.34, 1.05)	*F*_(1, 328)_ = 12.52^***^	0.41 (0.05, 0.77)
Overall quality of life	*F*_(1, 312)_ = 0.05	*F*_(1, 365)_ = 11.28^**^	0.72 (0.37, 1.08)	*F*_(1, 373)_ = 15.16^***^	0.74 (0.37, 1.11)
General health	*F*_(1, 281)_ = 0.21	*F*_(1, 341)_ = 17.45^***^	0.70 (0.34, 1.06)	*F*_(1, 351)_ = 8.41^**^	0.62 (0.26, 0.98)
**HADS**					
Anxiety	*F*_(1, 253)_ = 0.21	*F*_(1, 315)_ = 15.33^***^	−0.73 (−1.09, −0.38)	*F*_(1, 339)_ = 17.19^***^	−0.51 (−0.87, −0.15)
Depression	*F*_(1, 277)_ = 0.15	*F*_(1, 336)_ = 24.46^***^	−0.97 (−1.33, −0.60)	*F*_(1, 356)_ = 13.43^***^	−0.60 (−0.97, −0.24)
**DUREL**					
Organizational religiosity	*F*_(1, 215)_ = 0.55	*F*_(1, 263)_ = 0.58	0.08 (−0.27, 0.42)	*F*_(1, 270)_ = 0.11	0.14 (−0.22, 0.49)
Non-organizational religiosity	*F*_(1, 230)_ = 0.005	*F*_(1, 289)_ = 4.29^*^	0.31 (0.04, 0.66)	*F*_(1, 297)_ = 0.26	0.14 (−0.21, 0.50)
Intrinsic religiosity	*F*_(1, 199)_ = 0.40	*F*_(1, 236)_ = 0.96	0.07 (−0.27, 0.42)	*F*_(1, 241)_ = 0.72	0.14 (−0.04, 0.66)
Overall DUREL	*F*_(1, 192)_ = 0.30	*F*_(1, 223)_ = 2.06	0.16 (−0.19, 0.50)	*F*_(1, 227)_ = 0.53	0.20 (−0.16, 0.55)

*Post, post-intervention; FU, 6-month follow-up; d, Cohen's d; WHOQOL-BREF, World Health Organization Quality of Life–BREF; HADS, Hospital Anxiety and Depression Scale; DUREL, Duke University Religion Index*.

* *p < 0.05*,

** *p < 0.01*,

*** *p < 0.001*.

Additional mixed-model analyses showed that the type of work (i.e., internal vs. external) was not a significant covariate for WHOQOL-BREF domains and facets (*F*-values from 0.019 to 1.563; all *p*s > 0.05).

#### Secondary Outcomes: Depression and Anxiety (HADS), Religiosity (DUREL)

A significant group × time interaction effect was found for both HADS subscales, Depression [*F*_(2,247.44)_ = 12.52; *p* < 0.001] and Anxiety [*F*_(2,167.70)_ = 12.76; *p* < 0.001], and statistically significant differences between groups were found at post and 6-month follow-up, showing lower scores in the MBHP group compared with the WL group for both depression and anxiety subscales (Table 4). At pre-intervention, 32 (18.9%) and 52 (30.8%) participants scored above HADS-D and HADS-A cut-off (≥8), whereas at post-intervention, 20 (16.2%) and 27 (22.3%) officers scored above the cut-off, respectively. When considering the MBHP group, 16 (18.2%) and 29 (32.9%) valid responses were above HADS-D and HADS-A cut-off scores at pre-intervention, and 3 (4.7%) and 8 (12.7%) at post-intervention, respectively. Type of work was not a significant covariate for depression [*F*_(1,163.46)_ = 0.13; *p* > 0.05] and anxiety [*F*_(1,162.30)_ = 0.05; *p* > 0.05] subscales.

For DUREL, results showed only a significant interaction effect on non-organizational religiosity subscale [*F*_(2,241.62)_ = 3.51; *p* < 0.05]. Specifically, between-group comparison revealed statistically significant differences at post where the MBHP reported higher scores compared with the WL group (Table 4). Type of work (i.e., internal vs. external) was not a significant covariate for DUREL measure (*F*-values from 0.023 to 0.867; all *p*s > 0.05).

### Mediators for WHOQOL-BREF Increase After MBHP (Pre-to-post Intervention)

**Figure 2:**
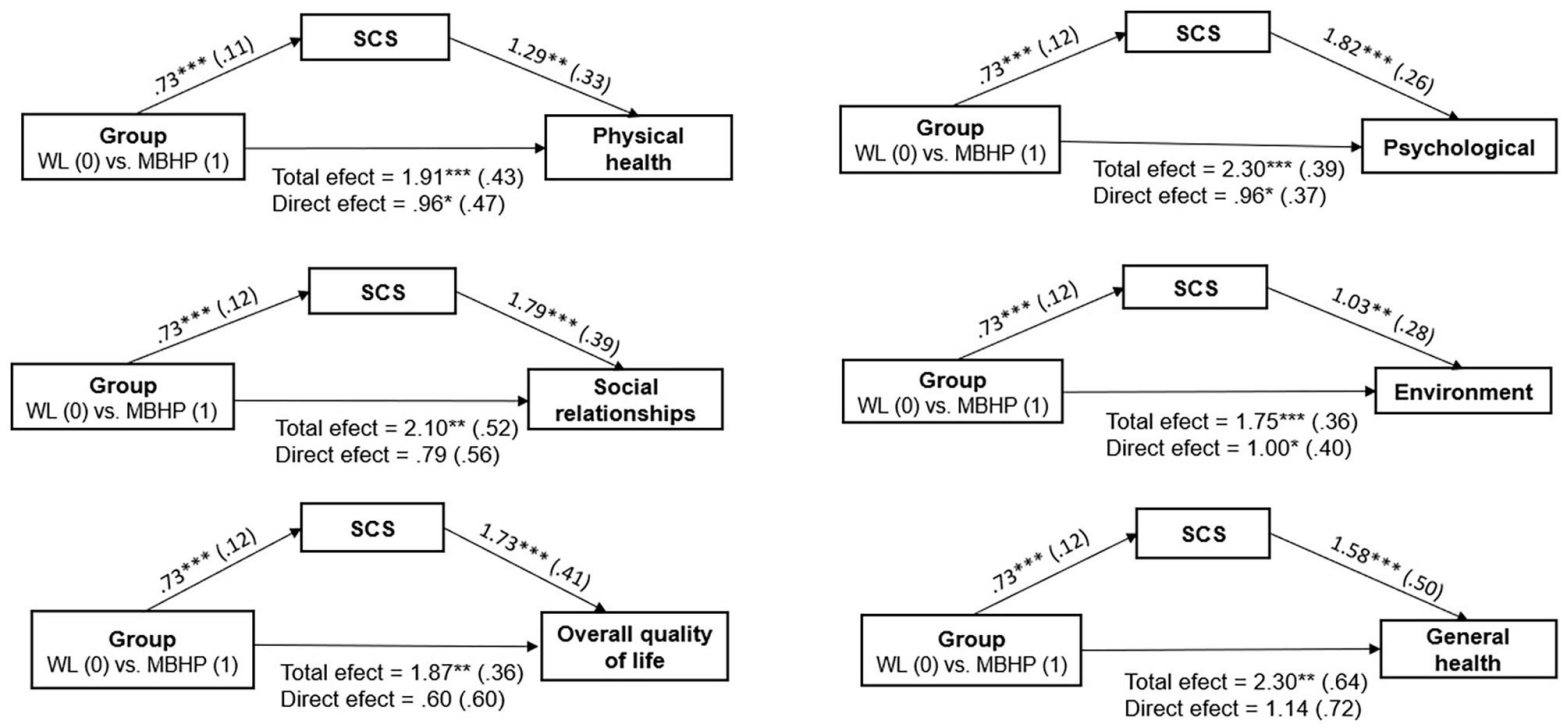
Mediation analyses for pre-to-post changes on quality of life (WHOQOL-BREF). SCS, Self-Compassion Scale. All coefficients represent unstandardized beta coefficients (standard errors in parentheses). Mediators and dependent variables are pre-to-post changes. *p < 0.05; **p < 0.01; ***p < 0.001.

Mediation results showed a significant indirect effect of MBHP on the change of QoL outcome through the change of self-compassion scores (SCSs) from pre-to-post intervention (Figure 2). Specifically, the total score of the SCS remained as the only significant mediator for pre-to-post changes on physical health [b = 0.94 (0.33), Ba CI 95% [0.34, 1.70]; *R*^2^ = 0.25], psychological [b = 1.33 (0.32), Ba CI 95% [0.78, 2.03]; *R*^2^ = 0.47], social relationships [b = 1.31 (0.37), Ba CI 95% [0.67, 2.16]; *R*^2^ = 0.26], environment [b = 0.75 (0.26), Ba CI 95% [0.29, 1.32]; *R*^2^ = 0.26], overall QoL [b = 1.27 (0.40), Ba CI95% [0.54, 2.18]; *R*^2^ = 0.22], and general health [b = 1.15 (0.38), Ba CI 95% [0.45, 1.89]; *R*^2^ = 0.18].

### Moderators for WHOQOL-BREF Increase After MBHP

Moderation was demonstrated by a significant interaction effect for general health facet, indicating that the relationship between the group and the pre-to-post change on the general health score was independently moderated by the pre-to-post change in MAAS [b = 1.28 (0.61), 95% CI [0.67, 2.49], t = 2.09, *p* < 0.05] and spirituality scores (WHOQOL-SRPB-BREF) [b = 0.098 (0.04), 95% CI [0.012, 0.18], t = 2.25, *p* < 0.05]. Specifically, increases on MAAS [b = 3.64 (1.02), 95% CI [1.63, 5.66], t = 3.59, *p* < 0.01] and spirituality (WHOQOL-SRPB-BREF) [b = 0.3.26 (0.86), 95% CI [1.55, 4.96], t = 3.78, *p* < 0.01] at post significantly moderated the group effect for general health. Sex, age, number of sessions, and changes in self-compassion and decentering scores were not yielded as significant moderators.

## Discussion

To the extent of our knowledge, we demonstrated the first empirical evidence grounded on a RCT that among police officers, an 8-week MBI (1) enhances all QoL domains and facets, (2) reduces depression symptoms, (3) decreases anxiety symptoms, (4) maintains QoL and mental health benefits after 6 months, (5) increases non-organizational religiosity, (6) has self-compassion as an important mechanism of action which mediates the improvement in all QoL domains and facets, and (7) is feasible in the Brazilian police environment.

### Primary and Secondary Outcomes

Participants randomized to receive MBHP demonstrated greater levels of self-reported QoL at post-intervention than the WL group across its multiple domains and facets with medium to large effect sizes. Notably, at 6-month follow-up, the difference between groups across all QoL domains and facets remained significant indicating medium effect sizes. As expected, the psychological domain was the one that showed the largest effect size magnitude between groups both at post-intervention and at 6-month follow-up. These findings are consistent with Grupe et al.'s (68) single-arm study with 30 LEOs who exhibited increased psychological well-being after 8 weeks of MT and with Christopher et al.'s (66) pioneering single-arm pilot (*n* = 43) that reported increased LEO global mental health following MBRT. Noteworthy, a study by the UK College of Policing suggests that online mindfulness resources improve officers' well-being (103). Our results are in accordance with the literature that indicates MT's positive impact in QoL and well-being across different populations (55, 81, 104), including military and war veterans (58, 62, 105, 106).

MBHP presented efficacy to decrease depression symptoms at post-intervention and at 6-month follow-up, displaying large and medium effect sizes, respectively. These results are in line with Grupe et al. (68) pre–post pilot findings that exhibited a trend level decrease on depression symptoms with medium effect size after MT. Individuals allocated to receive MBHP also showed reduced levels of anxiety when compared with the control group both at post-intervention and at 6-month follow-up with medium effect sizes. While Christopher et al.'s (67) rigorous RCT that enrolled 61 LEOs did not find a significant reduction on anxiety symptoms, the authors stated that the small sample size and type II error could explain the findings given that the trial main aim was to explore MBRT feasibility and acceptability. Our results resonate with the aforementioned Grupe et al. pilot (68) that showed a salient reduction in anxiety symptoms after MT which persisted after 5 months. Furthermore, a growing body of evidence denotes MT's declining effects on depression symptoms among military personnel (107–110).

Whereas, the scientific literature is still not clear about how long MBI effects last, its psychological benefits tend to decrease over time (111, 112). Previous studies with MBIs and police personnel mental health follow-up have mixed results (67, 68). Persistent salutary effects of MHBP after 6 months should be highlighted; however, the study design does not illuminate its longstanding impact as prolonged follow-up was not performed. The WL within-group significant reductions in QoL psychological domain after 8 weeks and the increment in depression symptoms after 8 weeks and at 6-month follow-up (Table 3) could be explained as a consequence of dealing with new stressors intrinsic to the police activity without mindfulness skills or due to the nocebo effect and frustration, disappointment, and anger about not being offered MBHP immediately (113).

Police officers' QoL and mental health impairment represented in the baseline assessment lower scores when compared with populational national and international samples might have contributed to the intervention impact (85, 114–116). For instance, the poor mental health of our non-clinical sample is expressed by the average score of 7.06 on HADS-Anxiety (Table 3), close to the ≥8 cut-off score that presents sensitivity and specificity for anxiety disorders of ~0.8 (117). Exposure to extensive criminality, low wages, long and irregular shifts, inadequate training, lack of equipment, corruption, hostile public image, bureaucracy, and rigid hierarchy, in addition to hazing, discrimination, sexism, and racism within departments, may represent Brazilian police work-related stressors that possibly explain these findings (6, 23, 118, 119). Inasmuch as organizational conditions affect the Police Institution as a whole, it explains why the covariate analysis showed that internal and external police officers equivalently benefit from the intervention. Brazilian police scientific literature de facto demonstrates that stress levels are similar between officers exerting operational or administrative roles (120, 121), in line with international data pointing that organizational stressors may be more challenging than operational experiences (122).

The significant interaction effect and difference between groups on non-organizational (private) religiosity at post-intervention can be attributed to the possibility that many participants considered mindfulness meditation a private religious activity and/or MT led participants to increase private religious activities such as other types of meditation, prayer, etc. MBHP, such as MBSR, is a secular behavioral medicine intervention that teaches skills addressed to reduce universal human suffering where spiritual themes are not explicitly explored. That being said, MBIs can enhance transcendence and awareness of interconnectedness in which oneself is not seen as separate from everyday activities, other people, or the world (123); thus, studies consistently have been suggesting that it might lead to increments in spirituality/religiosity (56, 124–126). MBHP components of values clarification and compassion/loving-kindness practices for oneself and others potentially contributed to this finding. It is also important to mention that Brazil is a highly religious country (predominantly catholic) where 90% of the population identify with a spiritual or religious group (127), and in our sample, that proportion was ~80%.

### Mediation and Moderation

The hypothesis that self-compassion could mediate QoL improvement was confirmed for all QoL domain and facets. Self-compassion has empirically shown to mediate MBI's effects on mental health and well-being (128–133). It is associated with a wide variety of positive outcomes related to psychological well-being and QoL (e.g., life satisfaction, positive affect, social connectedness, flourishing) (48, 134–137) and inversely associated with psychopathology (i.e., depression, anxiety, stress, suppression of unwanted thoughts, self-criticism, shame, anger) (137–141). Emotional regulation deficits and experiential avoidance are linked to depression, anxiety, and a lower QoL (142, 143). Denial, suppression, and avoidance of negative emotions are emotional regulation strategies commonly used by officers and those who are able to identify their feelings and be present to moment-to-moment experience show better mental health (144–146). The police toughness culture combined with exposure to uncontrollable situations, violence, and potentially traumatic situations results in high levels of shame, guilt, isolation, self-critical thinking, anger, and trauma-related disorders (2, 11, 146–148); therefore, self-compassion emerges as a crucial skill to be learned and cultivated. First-generation MBIs (i.e., MBSR, MBCT) did not explicitly teach self-compassion skills, but they are interwoven into the mindfulness instructions (e.g., “whenever you notice that the mind has wandered off, bring it back with gentleness and kindness”) (149, 150). In addition to implicit teaching, MBHP curriculum (73, 76) includes didactive teaching on compassion (e.g., fears of compassion), formal practices of compassion/loving kindness for oneself and informal practices (i.e., attention for self-compassion in routine activity), providing explicit opportunities for inner compassion cultivation.

Mindfulness trait and spirituality group effect moderation for WHOQOL-BREF general health facet pre–post changes are consistent with the literature that associates both characteristics with self-regulated behavior (151–153) and better health (154–157) in a variety of settings, including policing (69, 146, 158). Alsubaie et al.'s systematic review of MBCT and MBSR (159) concluded that global change in mindfulness are linked to better health outcomes. Historically, it should be remembered that MBSR—the first MBI—was developed in 1979 for people with chronic health conditions with the intention to create a vehicle for the effective training of medical patients in relatively intensive mindfulness meditation and its immediate applications to stress, pain, and illness (77). The fact that the number of sessions attended did not moderate the effect on QoL is aligned with Carmody and Baer's review (160) demonstrating no significant correlation between number of in-class MBSR hours and mean effect size. Moreover, our research group had previously suggested that an abbreviated MBI (four sessions) may have a similar efficacy to a standard MBI (eight sessions) in a non-clinical population (161).

### Feasibility

The findings indicate that MBHP is feasible among Brazilian police officers. Randomization process was successful considering that baseline characteristics were similar in both groups. Initial dropout rate was low, and most of the participants accepted the randomization and completed the assessments. MBI's “completion” has been defined as attending four or more sessions (112). In view of these criteria, our completion rate was high and comparable with previous MBI studies (162), including within the police (67, 68).

### Strengths and Limitations

The empirical results reported herein should be considered in the light of some limitations. Our sample was composed predominantly of female officers (Table 2) working at two of the most violent cities in the world (119). We used a waitlist control design rather than an active control intervention. While there are ethical advantages to a waitlist design because it allows for the provision of care to research participants who are seeking help while permitting a non-intervention evaluation, such design may overestimate intervention effects (163). Finally, single data entry may be associated with errors within the registry and a MIXED procedure with a random intercept for subject was conducted but test for random slope was not considered. The POLICE study has several strengths including the relevance and potential societal impact of applying MBIs within the police environment and its design—a multicenter RCT with follow-up assessment. RCTs are the “gold standard” in evidence-based medicine and the only type of study able to establish causation. Other key strengths are the sample size that allowed us to detect differences between groups and the focus on mental health/quality of life promotion and prevention of psychological suffering. Given mental illness' emotional, physical, economic, and social cost, a balanced approach between early intervention strategies and treatment is needed, consonant with preventive medicine and positive psychiatry (70, 164).

## Conclusion and Future Directions

The POLICE study makes an important contribution to the emerging field of MT for police mental health and QoL. Although on its infancy, results from the first trials are promising. Qualitative studies are needed to understand officers' attitudes, feelings, and behaviors toward MT in greater depth (e.g., language attunement; potential obstacles and resistances related to police norms and customs; impact on family life). Occupational health literature suggests that physical exercise, MBIs, cognitive-behavioral therapy, and change in organizational practices promote mental health and well-being with no particular superiority, although more rigorous evaluations are needed (165). Future police RCTs should use active control interventions. Long-term follow-up, cost-effectiveness data, cross-cultural research, and replication on male samples will play a pivotal role for the field expansion. Complementary neurobiological, cognitive, behavioral, and psychometric measures addressing mechanisms (e.g., attentional/cognitive control, emotional regulation, interoceptive awareness, rumination) and a wide scope of outcomes, encompassing health and well-being; workplace (e.g., interpersonal relationships, absenteeism, safety, leadership, return on investment); societal (e.g., mental capital, ethics, impact on civilians); and effective policing (e.g., impulse control, working memory, task performance) perspectives should be on the research agenda. Importantly, if mindfulness and loving-kindness meditation could reduce implicit racial bias (166, 167), its impact in decreasing officers' stereotype-biased judgments and behaviors would warrant further investigation. “Stepped-care” and “low intensity–high volume” approaches (168) can increase accessibility and facilitate the nurturing of “Mindful Police Departments.” Evidence-based programs designed for cultivation and embodiment of self-compassion skills such as mindful self-compassion (169) and compassion cultivation training (170) or the interweaving of self-compassion practices in MT are auspicious. Rigorous research will inform and guide procedures, public policy decision-making, and systematic real-world implementation of MT for Police Institutions around the globe, nourishing the conditions for officers' physical, emotional, and mental fitness, and contributing to the judicious and mindful use of police power and authority, which could benefit society as a whole.

## Data Availability Statement

The raw data supporting the conclusions of this article will be made available by the authors, without undue reservation.

## Ethics Statement

The study was approved by HCPA and UNIFESP Research Ethics Committees under number 60406416.9.1001.5327. The patients/participants provided their written informed consent to participate in this study.

## Author Contributions

MT, MD, and NR were responsible for manuscript writing, co-conceptualized, designed the study, and obtained funding. DC were responsible for statistical analysis and manuscript writing. MD, SA, and KC coordinated the study at São Paulo center. SA was particularly involved in the implementation and conduction of the project at this site. MT and NR coordinated the study implementation and conduction at Porto Alegre center. KC and AW were responsible for data curation. JG-C and ZS-O commented and critically reviewed the manuscript for important intellectual content. All authors read and approved the final manuscript.

## Conflict of Interest

The authors declare that the research was conducted in the absence of any commercial or financial relationships that could be construed as a potential conflict of interest.
